# The Effect of L-Carnitine on Critical Illnesses Such as Traumatic Brain Injury (TBI), Acute Kidney Injury (AKI), and Hyperammonemia (HA)

**DOI:** 10.3390/metabo14070363

**Published:** 2024-06-27

**Authors:** Bharti Sharma, Lee Schmidt, Cecilia Nguyen, Samantha Kiernan, Jacob Dexter-Meldrum, Zachary Kuschner, Scott Ellis, Navin D. Bhatia, George Agriantonis, Jennifer Whittington, Kate Twelker

**Affiliations:** 1 Department of Surgery, NYC Health and Hospitals, Elmhurst, 79-01 Broadway, New York, NY 11373, USA; nguyenc15@nychhc.org (C.N.); kushnerz@nychhc.org (Z.K.); elliss5@nychhc.org (S.E.); bhatian1@nychhc.org (N.D.B.); agriantg@nychhc.org (G.A.); harrisj20@nychhc.org (J.W.); twelkerk1@nychhc.org (K.T.); 2 Icahn School of Medicine at Mount Sinai, New York, NY 10029, USA; lee.schmidt@mountsinai.org (L.S.); jacob.dexter-meldrum@icahn.mssm.edu (J.D.-M.); 3 Touro College of Osteopathic Medicine–Harlem, New York, NY 10027, USA; kiernans1@nychhc.org

**Keywords:** L-carnitine, traumatic brain injury, acute kidney injury and hyperammonemia

## Abstract

L-carnitine (LC) through diet is highly beneficial for critical patients. Studies have found that acetyl-L-carnitine (ALC) can reduce cerebral edema and neurological complications in TBI patients. It significantly improves their neurobehavioral and neurocognitive functions. ALC has also been shown to have a neuroprotective effect in cases of global and focal cerebral ischemia. Moreover, it is an effective agent in reducing nephrotoxicity by suppressing downstream mitochondrial fragmentation. LC can reduce the severity of renal ischemia-reperfusion injury, renal cast formation, tubular necrosis, iron accumulation in the tubular epithelium, CK activity, urea levels, Cr levels, and MDA levels and restore the function of enzymes such as SOD, catalase, and GPx. LC can also be administered to patients with hyperammonemia (HA), as it can suppress ammonia levels. It is important to note, however, that LC levels are dysregulated in various conditions such as aging, cirrhosis, cardiomyopathy, malnutrition, sepsis, endocrine disorders, diabetes, trauma, starvation, obesity, and medication interactions. There is limited research on the effects of LC supplementation in critical illnesses such as TBI, AKI, and HA. This scarcity of studies highlights the need for further research in this area.

## 1. Introduction to L-Carnitine

Carnitine (also known as β-hydroxy-γ-*N*-trimethyl-aminobutyric acid) [1] is a quaternary amine compound [2,3]. Its biologically active stereoisomer is L-carnitine (LC) [4]. It was first discovered in 1905 by Gulewitsch, Krimberg, and Kutscher in a muscle extract [2,3], followed by an exploration of its structural and optical attributes in 1927 [5]. LC can stimulate oxidation of fatty acids (FA) and transport activated long-chain FA for β-oxidation in mitochondria [6]. For over a century, researchers have studied LC [2]. LC is present in all domains of life [2,3] and is essential for human health [7,8]. In eukaryotes, LC can be produced endogenously, whereas in prokaryotes, it can be catabolized [9]. Its availability is limited in plants, whereas food obtained from animals is rich in LC [1]. Diet provides ~75% of carnitine in humans [10]. Around 99% of LC is intracellular [11]. Some of the LC-rich food items are red meats such as beef and lamb, fish, poultry, and milk [12,13]. In omnivores, a major source of LC is dietary ingestion [5,9]. Dietary LC has been reported with a bioavailability (BA) of ~63% to 75% [14]. LC in the vegetarian population has a BA of ~ 66% to 86%, whereas in red meat eaters, BA is ~54% to 72% [15]. In most cases, an adult’s recommended average daily carnitine intake is 20–200 mg, which is met through food and internal production. An LC dose of ~3 g/day can result in abdominal cramps, vomiting, nausea, diarrhea, and a fishy body odor [16,17].

LC is a branched non-essential amino acid and is synthesized from lysine and methionine in the liver, brain, and kidney [11]. It was found in bovine muscle extract and is a vitamin-like substance or micronutrient that serves as an essential component for body function and metabolism [18]. The mammalian pathway utilizes enzymatically methylated protein-bound lysine as a post-translational modification of protein synthesis to form trimethyl lysine [19]. Unabsorbed LC is degraded by microbes in the intestinal tract [4]. Synthesized metabolites such as TMAO and gamma-butyrobetaine [20] can result in cardiovascular diseases (CVD) [21,22,23]. The highest concentration of LC is present in skeletal and cardiac muscle [4]. Studies have reported two different LC deficiency conditions: primary carnitine deficiency (PCD) and secondary carnitine deficiency (SCD) [24]. PCD is an autosomal recessive disorder [25,26]. LC depletion is common in patients with advanced LC and in individuals with genetic abnormalities. [27,28]. Lack of LC cellular transporters such as OCTN2 may result in PCD and can enhance the loss of renal LC [25,26]. Due to impaired renal absorption, free LC is excreted through the urine [29,30]. Overall, it results in LC depletion [31]. Characteristics of SCD involve excess excretion of LC in acyl-carnitine form [32,33]. Peritoneal dialysis, metabolic disorders, malabsorption, diet poor in LC, and Fanconi syndrome (free carnitine loss through renal tubules) are some of the triggering factors in SCD [34,35]. LC as a supplement is highly beneficial in LC deficiency syndromes [36]. 

LC is a non-protein amino acid [19,37]. Carnitine acetyltransferase (CAT) helps in the formation of ALC and propionyl-LC (PLC) [38,39]. CAT can be found in peroxisomes and mitochondria. It is highly expressed in liver and heart tissues [38,39]. Acetylation of LC allows ALC to cross the blood–brain barrier [40]. LC can remove unmetabolized acyl groups from mitochondria by utilizing free coenzyme A [41]. The kidney plays an essential role in the excretion of acylcarnitine (AC). AC promotes LC depletion by inhibiting carrier-mediated reabsorption of LC in renal tubules [41]. LC and its acylated derivatives constitute a carnitine pool (CP). In cellular organelles like peroxisomes and mitochondria, CP facilitates fatty acid β-oxidation (FAO) [35,42]. A diverse group of mitochondrial-derived metabolites is represented by CP. Blood concentration of these metabolites can help in the diagnosis of several disorders [33,43,44]. Oxidation of mitochondrial FA due to deficiency of carnitine palmitoyltransferase (CPT) is not uncommon [45]. ALC can suppress mitochondrial malfunction, promote neuroprotective effects in brain cells, repair neurons, reduce apoptosis, and enhance energy metabolism [40]. It can modulate the pyruvate dehydrogenase complex (PDH) and the intramitochondrial acetyl-CoA/CoA ratio. Thus, it is essential for glucose metabolism, fatty acid metabolism, and the obtainment of acetyl-CoA [46,47]. It is an important cofactor of carnitine palmitoyltransferanse 1 (CPT1) [46,47]. CPT-I and CPT-II help in the transportation of long chains of FA (carbon chain length = 10) across outer- and inner-mitochondrial membranes [48]. Enzyme activity measurement of CPT-I and CPT-II can be used as a powerful diagnostic tool for LC and mitochondrial-associated diseases [45]. LC is an obligatory cofactor that acts as a shuttle for acetyl groups [49]. It facilitates the transportation of long-chain FA as acylcarnitine esters across the mitochondrial membrane [49]. Oxidation of FA through the mitochondrial β-oxidation system is necessary to meet the energy requirements of the body [50]. During fasting, it provides ~80% of the total energy [50]. In tissues like cardiac and skeletal muscle, long-chain FA is an important source of respiratory fuel [41].

LC is a safe dietary compound and does not exhibit any toxicity, carcinogenic activity, or chromosomal abnormalities [51]. As a food supplement, it can be easily tolerated [52]. According to a double-blind, cross-over trial conducted by Vecchiet et al. (1990), LC when administered orally (2 g of LC or a placebo) can boost oxygen uptake and power output in young men [53]. It is essential for healthy mitochondrial function [54] and fuel-sensing [49]. It has protective functions such as the elimination of toxic metabolites, the protection of cellular membranes, and the prevention of FA accumulation [54]. It plays an important role in energy production by facilitating the entry of FA into mitochondria [55]. Disruption in FA oxidation can alter total plasma LC concentration. For example, it can elevate the acyl/free LC ratio, or it can lower total plasma LC concentration [41]. LC can mediate oxidation of peroxisomal FA, removal of excess acyl groups, metabolism of branched-chain amino acids, buffering of the acyl-coenzyme A (CoA)/CoA ratio, and mitochondrial oxidation of long-chain FA using specialized acyltransferases [56]. It preserves the integrity of the mitochondrial electron transport chain by acting as a mitochondrial antioxidant [57]. It is transported across intestinal cell membranes using active and passive transport [4]. Sakurauchi et al. (1998) conducted a randomized trial of 107 patients. For 12 weeks, 500 mg oral LC was administered to patients. In patients with hemodialysis, improvement in dialysis-associated muscle symptoms has been reported [58]. In another study evaluating LC’s safety profile, a sub-chronic toxicity test lasting 90 days on rats found that administering up to 50,000 ppm LC in their diets did not result in any treatment-related changes in mortality, gross pathology, hematology, ophthalmology, or histopathology. It was also observed that LC did not show any carcinogenic activity in different bacterial strains at doses up to 5000 g per plate, with or without metabolic activation. Additionally, it did not cause chromosomal abnormalities in human cells. These studies’ findings confirm that using LC as a dietary source is safe [59].

LC is crucial for β-oxidation, as it imports activated long-chain FA into mitochondria from cytoplasm [36]. It can downregulate gene expression associated with oxidative stress like caspase-3, Bax, and Bcl-2 [57]. It contributes toward the regulation of transcriptional factors like PPAR-γ, nuclear-factor-erythroid-2-related-factor-2 (Nrf2), NF-κB, and PPAR-α and can affect levels of SOD, catalase (CAT), glutathione reductase (GR), and glutathione-peroxidase (GSH-Px) [57]. It can upregulate the expression of sirtuins, thioredoxin, and heat shock proteins [57]. Mitochondria are known as the powerhouse of cells. Any mitochondrial abnormality can negatively influence energy production [60,61]. LC maintains metabolic flexibility by preserving mitochondrial-free CoA levels [54]. Its structure is similar to choline, and it has no role in neurotransmission [62]. Treatment with LC can improve metabolism in ischemic heart disease and myocardial functionality [59]. Cardiac and skeletal muscles draw LC from plasma due to a lack of ability to synthesize it [4]. LC and its esters can combat oxidative stress and break down fat. Hence, LC can be present in conditions such as angina, weight loss, and heart failure [12]. LC plays a significant role in aging, cirrhosis, cardiomyopathy, malnutrition, sepsis, endocrine disorders, diabetes [59], trauma, starvation, medication interactions [63], osteoarthritis, infertility, dementia, obesity, athletic performance, improvement of sperm motility, and morphology. The roles of LC in various conditions are shown in Table 1 of this paper.

Though the beneficial effects of LC supplementation in critically ill patients have been researched and remain a topic of debate, the impact of LC specifically in traumatic brain injury (TBI), acute kidney injury (AKI), and hyperammonemia (HA) patients has not yet been well studied. Most trials have looked into chronically critically ill ICU patients whose serum carnitine levels are notably reduced and thus are most likely at risk of LC deficiency [71]. Limited studies have looked at the effects of LC supplementation on monitoring laboratory variables in patients with TBI. Dietary administration of LC is reported as beneficial to patients with critical illnesses. Although studies have evaluated the development of LC deficiency in critically ill patients, the effect of LC in these patients has not been fully studied [72]. Our goal is to bring forth studies investigating LC in critical illnesses like TBI, AKI, and HA.

## 2. L-Carnitine in Traumatic Brain Injury

According to the Centers for Disease Control and Prevention (CDC), in 2020, there were 214,110 recorded cases of TBI-related hospitalizations. In 2021, there were 69,473 TBI-related deaths, with an average of more than 586 TBI hospitalizations and 190 TBI deaths per day. Individuals aged 75 years and older were found to be particularly vulnerable to TBI-associated hospitalizations and deaths [73]. The pathophysiological response to TBI starts with a series of events that disrupt normal cell function and trigger inflammatory responses, oxidative stress, and mitochondrial dysfunction. LC has been demonstrated to improve mitochondrial dysfunction [74,75]. In carnitine synthesis, the terminal gamma-butyrobetaine hydroxylation reaction occurs strictly in the liver, kidney, and brain in humans [76].

Though a deficiency in LC is common in critically ill patients, it has not been well studied in patients with TBIs. ALC has been reported to provide neuroprotection in various animal models of global and focal cerebral ischemia [74]. Zanelli et al. (2005) found significantly reduced levels of lactate and inorganic phosphate, as well as elevated ATP and creatine-phosphate in the damaged brain tissues of animals that were administered ALC compared to the untreated group. This study suggests that ALC helps increase the brain’s energy production by promoting oxidative processes, which reduces anaerobic glycolysis and the accumulation of lactic acid [74]. Additionally, ALC may indirectly protect against oxidative stress by reducing tissue lactic acidosis. Following an injury to the brain, oxidative stress can lead to decreased pyruvate dehydrogenase activity during reperfusion. A study found that animals treated with ALC after cardiac arrest showed significant improvement in brain pyruvate dehydrogenase enzyme activity at 30 min and 24 h after reperfusion [74]. The same study also explored the role of ALC in inhibiting neural excitotoxicity, where rat cortical neurons in the presence of ALC inhibited acute and delayed cell death after exposure to an excitotoxic glutamate antagonist.

**Figure 1:**
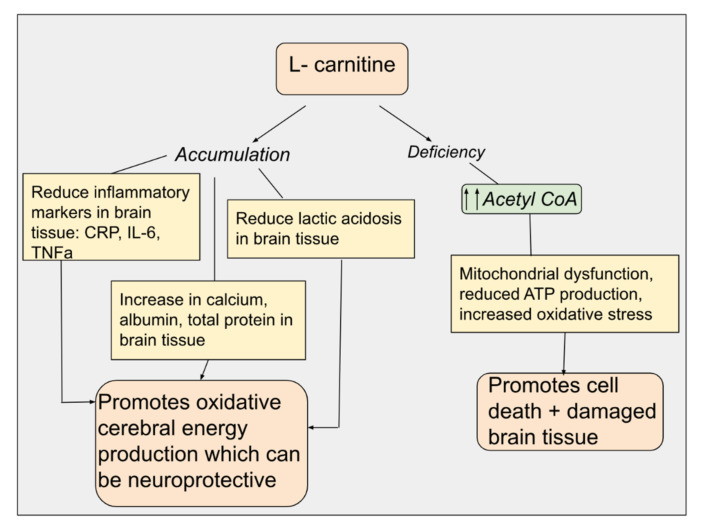
Mechanisms of action in neuroprotection by L-carnitine.

The role of mitochondria is central to brain injury. Specifically, for TBI, it is suspected that abnormal mitochondrial function is also observed in TBI patients. Post-traumatic pathways that promote cell death such as excitotoxicity and oxidative stress can have significant effects on mitochondrial dysfunction, as seen in adult animal and human studies [77]. Since LC contributes to antioxidant reserve and optimizes mitochondrial function, some studies have shown that LC administration in the acute phase of TBI can result in neurobehavioral function improvement and reduced levels of injury in immature rats [75]. In a recent study, LC therapy for 7 days was found to reduce the occurrence of cerebral edema and also to decrease long-term neurological issues by enhancing neurobehavioral and neurocognitive function in patients following TBI. This is due to LC’s ability to increase ATP levels and decrease lactate levels in brain tissue after an ischemic event. LC can also reduce post-ischemic markers of oxidative stress in brain tissue and cerebrospinal fluid, inhibit mitochondrial permeability transition, and decrease acute and chronic cell death following an insult. LC likely improves cerebral energy metabolism. LC is beneficial as a neuroprotective drug in patients with cerebral inflammatory states and edema, such as those resulting from TBI. Figure 1 of this paper summarizes the mechanism of action of LC as a neuroprotective as well as a neurotoxic agent. Deficiency in LC may contribute to neurologic tissue damage with increased cell death due to accumulation of acetyl-CoA, which potentially affects mitochondrial function leading to a reduction in ATP production. Alternatively, an accumulation of LC can lead to the neuroprotective role by reducing inflammatory markers and lactic acid build-up and increasing calcium, albumin, and total protein in brain tissue. Collectively, this promotes cerebral energy production, which can be neuroprotective.

Neuron-specific enolase (NSE) is an inflammation marker in patients with TBI. In a clinical study conducted by Mahmoodpoor et al. (2018), 40 patients were randomized into two groups, with (2 g/day LC for one week) and without (a placebo). LC was shown to improve neurobehavioral function, with no improvement in mortality, degree of cerebral edema, and unsuppressed NSE levels [71,75]. In a study conducted by Bounes et al. (2018), 95% of the patients were reported with lower plasmatic free LC concentration with a median value of 18 μmol/L, proving the suppressed level of LC in patients with severe TBI [78]. In a retrospective study conducted by Daia et al. (2021), 60 patients with unresponsive wakefulness syndrome (UWS) after severe TBI were divided into two groups: with and without neuroprotective treatment. LC and some other neuroprotective agents alongside standard treatment were administered. Recovery from UWS was more rapid in the LC group than in controls, and it was shown to improve progressively [79].

## 3. L-Carnitine in Acute Kidney Injury

Due to its high energy utilization and critical role in filtering toxic as well as non-toxic metabolites, the kidney is vulnerable to injury precipitated by ischemia, mitochondrial dysfunction, and oxidative stress. In healthy kidneys, renal tubular cells are rich in mitochondria to meet the high ATP requirements demanded by the absorption of high volumes of ultrafiltrates and solutes, and these mitochondria rely on oxidative phosphorylation and fatty acid β-oxidation [80]. The electron transport chain (ETC) produces ROS as a byproduct, and a balance between promoting metabolism and scavenging these free radicals is critical for mitochondrial function. Enhanced ROS levels can lead to mitochondrial dysfunction, inflammation, cell death, and AKI. Nuclear factor kappa B (NFκB), inflammasomes, pro-inflammatory cytokines, and interleukins are some of the contributing factors [80]. Mitochondrial dysfunction associated with cell death and severity of kidney injury plays a critical role in critically ill patients. Broadly, three categories of AKI can be linked to the disruption of mitochondrial metabolism and ROS scavenging: nephrotoxic (due to medications such as chemotherapeutic agents), ischemia/reperfusion injury (such as in trauma, vascular, or renal transplant patients), and cytokine-mediated/other pro-inflammatory models of AKI. Together, these three categories encompass almost all causes of AKI in critically ill patients.

LC, as previously discussed, is synthesized in vivo from L-lysine and L-methionine, mostly in the liver and kidney [81]. It acts as an essential co-transporter of long-chain FA into the mitochondrion for β-oxidation and introduction of those fatty acid byproducts into the citric acid cycle and then into the ETC for the production of ATP [82]. In otherwise healthy patients, the endogenous synthesis of LC from dietary FA is sufficient to maintain this transport of long-chain FA. However, in patients who are in a state of metabolic stress, supplementation may be required to maintain homeostasis [81]. In addition to its mechanism of action as a fatty acid co-transporter, LC has a protective effect on mitochondrial function via a direct antioxidant effect as a free radical scavenger, as well as via the promotion of endogenous antioxidant defenses, preventing the accumulation of end products of lipid peroxidation, which are toxic to renal tubular cells [83,84,85]. LC inhibits caspases 3 and 8, which are essential enzymes in the apoptotic pathway [81]. Such actions make LC a compelling molecule for supplementation in critically ill patients who are at risk for AKI. Here, we will discuss the renal protective effects of LC supplementation in various preclinical models of AKI.

### 3.1. L-Carnitine in Drug-Induced Models of Acute Kidney Injury

There are many drugs given to patients in the intensive care unit that are well known to be nephrotoxic. Several compounds have been well studied as rat models of nephrotoxicity, including the chemotherapeutic agents cisplatin and carboplatin and the aminoglycoside antibiotic gentamicin. Cisplatin and carboplatin lead to renal tubular cell apoptosis via oxidative stress-induced activation of the transcription factors, nuclear factor of activated T cells (NFAT3), and NFκB and pro-inflammatory cytokine tumor necrosis factor-α (TNF-α) and monocyte chemoattractant protein-1 (MCP-1) [86]. Similarly, gentamicin leads to oxidative injury and inhibition of mitochondrial oxidative phosphorylation [87]. These drugs share common pathways of nephrotoxicity, as seen in many medications administered to hospitalized patients.

LC acts to mediate drug-induced AKI in rat models. In a 2002 study of gentamicin-induced renal injury in rats, LC administered 4 days before gentamicin was shown to significantly decrease serum urea, increase creatinine clearance, and decrease the severity of histopathological changes at low and high doses on day 12 compared to rats who only received gentamicin [87]. Additionally, rats given a high dose (200 mg/kg/d) of LC had significantly less proximal tubular necrosis than rats who received only gentamicin or gentamicin along with low doses of LC (40 mg/kg/d).

A 2009 study of ALC administered to rats who had received nephrotoxic doses of cisplatin showed suppressed caspase 3 and 9 activity, improved kidney function, and decreased cell death compared to rats who received only cisplatin [88]. These findings have been replicated in rats receiving cisplatin and carboplatin. A 2014 study of LC in rats with carboplatin-induced renal injury found that LC prevented renal tubular cell apoptosis via AMPK-mediated PPAR-α activation in proximal renal tubular epithelial cells, and that rat renal tubular cells (RTCs) that received LC pretreatment had decreased NFκB transcriptional activity as well as decreased production of TNF-α, ICAM-1, and MCP-1 compared to RTCs that did not receive LC [86]. This decrease in nephrotoxicity after LC treatment is hypothesized to be linked to reduced downstream mitochondrial fragmentation and improved adaptation of mitochondria to high energy demands, as well as ROS scavenging by LC [80,86,89].

### 3.2. L-Carnitine in Ischemia-Reperfusion Models of Acute Kidney Injury

Renal ischemia disrupts the substrate’s availability for oxidative phosphorylation and restoration of flow leading to reperfusion injury due to the release of ROS at a rate that outpaces the cells’ ability to scavenge and convert them. This form of injury is referred to as ischemia-reperfusion injury. Depletion of ATP and activation of proteases, NO synthetases, and phospholipases result in activation of the apoptotic cascade and cell death [85]. Several markers have been identified as indicators of ischemia-reperfusion injury, including nitric oxide (NO), which is overproduced during ischemia-reperfusion and converted to free radicals; myeloperoxidase (MPO), which is released from neutrophil granules during phagocytosis; and MDA (malondialdehyde), which indicates levels of lipid peroxidation or oxidative deterioration of lipids [90,91].

Several studies have examined the role of LC or ALC for the prevention and amelioration of ischemia-reperfusion injury in cell and animal models. Pre- or co-treatment with LC or ALC in models of renal ischemia is associated with significantly milder histopathological changes, lower levels of MDA, MPO, and TNF-α, and improved glomerular filtration rate (GFR) and renal function compared to animals that underwent renal ischemia without LC [83,90,92,93,94,95]. These studies indicate that antioxidants such as LC/ALC may decrease oxidative stress caused by ischemia-reperfusion injury, thereby inhibiting apoptotic cell death. This antioxidant effect appears to be both due to direct ROS scavenging by LC as well as the promotion of endogenous antioxidant defenses such as lipid hyper peroxidation [83,91]. These studies indicate that LC and its derivatives have significant promise in decreasing the severity of renal ischemia-reperfusion injury.

### 3.3. L-Carnitine in Other Pro-Inflammatory Models of Acute Kidney Injury

**Figure 2:**
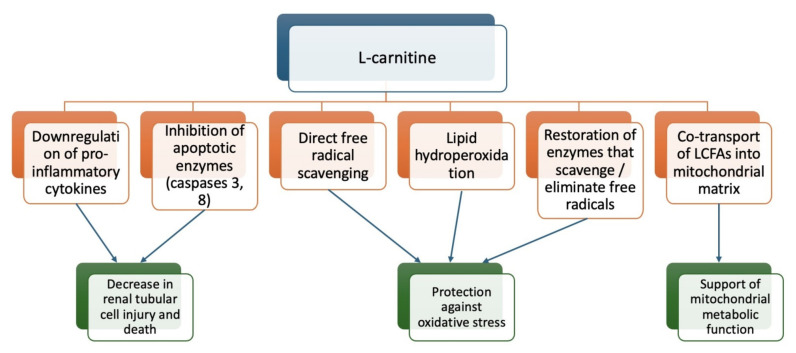
Mechanisms of action in renal protection by L-carnitine.

Other animal models for AKI include myoglobinuric models of rhabdomyolysis and acute tubular necrosis (ATN), induced by the administration of glycerol, and carbon tetrachloride (CCl_4_)-induced oxidative stress. Two studies of glycerol-induced myoglobinuric renal failure in rats showed that LC administration had a protective effect on renal tubular cells [84,94]. A 2006 study showed that LC significantly decreased renal cast formation, tubular necrosis, iron accumulation in tubular epithelium, creatinine kinase activity, urea levels, creatinine levels, and MDA levels and reversed depletion of GSH. It restored the function of superoxide dismutase (SOD), catalase (CAT), and glutathione peroxidase (GPx), which function to remove and scavenge free radicals [91,94]. Similarly, in a 2015 study, LC significantly decreased BUN, creatinine kinase levels, and the percentage of histopathologically injured glomeruli and tubules and significantly increased urine volume and cytochrome p450 activity compared to rats who received glycerol alone [84]. CCl_4_ serves as the final model of AKI to be discussed in this section. It leads to free radical damage that in turn leads to peroxidative degradation of endoplasmic reticulum membrane lipids. In a study of CCl_4_-induced oxidative stress in Wistar rats, ALC administration maintained activity of SOD, CAT, and GPx after CCl_4_ administration and maintained GSH levels (reducing glutathione, which detoxes ROS and serves as an essential co-factor for GPx activity) and MDA levels [91]. The level of LC in the serum can indicate kidney function, because the kidneys are the main organs responsible for the synthesis and metabolism of LC in animals. When kidney function declines, it can lead to reduced carnitine clearance and impaired excretion of ALC, resulting in LC deficiency. Taken together, these models provide further evidence that LC prevents lipid peroxidation, serves as a ROS scavenger, and enhances endogenous cellular antioxidative mechanisms. It has been suggested that LC supplementation or nutritional therapy may provide some benefits for patients with chronic renal failure. Figure 2 of this paper illustrates the role of LC in AKI.

A clinical study (NCT02664753) was started in 2018 and is estimated to be completed by 2025-04. The goal of this study is to compare the mortality rate of septic shock patients with renal insufficiency. LC will be administered to the treatment group as an adjunct therapy for 56 days, and the non-treatment group will not receive LC. This study is currently in phase 3 [96]. Mohammadi et al. (2017) conducted a clinical study to investigate the nephroprotective effects of LC. Patients with elective percutaneous coronary intervention (PCI) were included. Among 202 patients, the treatment group received 1 g of LC orally 3 times a day, 24 h before the catheterization procedure (3 g was given before PCI, and 2 g after PCI). Oral LC was reported to prevent elevation of neutrophil gelatinase-associated lipocalin (NGAL) in patients undergoing PCI showing nephroprotective effects of LC [97]. Another clinical study by Jafari et al. (2017) investigated the protective effect of LC in patients with delayed graft function (DGF). DGF is an early complication in patients with transplanted kidneys of a deceased donor. Fifty-six patients older than 14 years old were randomly assigned to treatment (LC) and non-treatment group (placebo groups). Three grams of oral LC were administered in three divided doses for four consecutive days. No difference in DGF incidence and no protective effects of oral LC in DGF patients were reported [98]. In 2023, Gennaro and his team aimed to explain the population pharmacokinetics (PK) of high-dose LC, test different estimates of kidney function, and evaluate the association of PK parameters with pre-treatment metabolites in explaining drug response for patients with septic shock. They collected 542 serum samples from 130 patients with septic shock. Based on allocated groups, LC (6 g, 12 g, or 18 g) or placebo was administered to patients. Patients with a high concentration of LC showed worse kidney function and were highly metabolically dysfunctional. If a patient with sepsis has high levels of acylcarnitines, the risk of disease severity and mortality is also higher, especially when no supplemental LC is provided to the patient [99]. In a controlled pilot cohort study of 48 children receiving prolonged continuous kidney replacement therapy (CKRT), the effect of LC on myocardial strain was investigated by Sgambat et al. (2021). Total (TC) and free plasma carnitine (FC) were measured in patients with AKI, followed by intravenous levocarnitine supplementation, 20 mg/kg/day. LC improved myocardial strain and was associated with the repletion of plasma carnitine in patients undergoing CKRT [100]. Some of the LC-related clinical trials are shown in Table 2 of this paper.

## 4. L-Carnitine in Hyperammonemia

A PubMed search was performed through January 2024 using the terms *HA*, *LC*, and *ammonia*, and references from articles obtained in the search were also screened for inclusion. Only articles studying the treatment of acute, emergent HA with LC were included. Articles that assessed long-term supplementation of LC to suppress or prevent HA were not included. Thirteen articles were found [101,102,103,104,105,106,107,108,109,110,111,112,113]. Of these articles, eleven had HA associated with the use of valproate, either as a high-dose therapy [101,102,104,105,106,107,109,110,111] or overdose [103,108,110].

Nine of these articles (Table 3) [101,102,103,105,106,107,108,109,110] presented case studies with initial ammonia levels ranging from 65–>700 μmol/L. LC was administered in varying forms, including IV and via NG tube, and additional treatments included continuous renal replacement therapy [103] and lactulose [102]. Final ammonia levels after treatment ranged from 22 to 58.13 μmol/L, and patients were described as returning to baseline status. One retrospective, system-wide, cohort study [104] of critically ill patients receiving valproate therapy assessed multiple therapies, including LC, for HA. Initial ammonia levels ranged from 42 to 70 μmol/L. Patients were given a median dose of 1500 mg/day LC, either IV or PO, after which ammonia levels were reduced to 28–45 μmol/L after 48 h. The final article [111] retrospectively reviewed 13 cases of HA and encephalopathy related to treatment with valproate or valproate overdose treated with LC in pediatric patients. The mean ammonia level of these patients was 231.7 μmol/L, with a maximum of 557 μmol/L, and all patients received IV LC ranging from 100 to 200 mg/kg by infusion over 30 min every 8 h until clinical improvement. None of these studies reported any adverse effects associated with LC.

The remaining two articles were a case study of an adult receiving chronic enteric tube feeding and being treated for seizures with phenytoin [112] and a case study of a chronic hemodialysis patient with hepatitis C infection [113]. The chronic enteric tube-feeding patient [112] presented with confusion and drowsiness and was found to have an ammonia level as high as 100 μmol/L. The patient was supplemented with 500 mg BID LC via PEG tube, the ammonia levels were reduced to 53 μmol/L, and the patient returned to baseline. The hemodialysis patient [113] presented with altered mental status and was found to have an initial ammonia level of 189 μmol/L, which increased to 215 μmol/L. Two grams of LC were administered, and within three hours, the patient’s mental status returned to normal. Ammonia levels measured the next day had decreased to 37 μmol/L. No LC-associated adverse effects were reported in these studies.

## 5. Toxicological Profile and Safety Concern

The American FDA has approved LC in powder, fluid, tablet, or capsule form for treating primary and secondary carnitine insufficiency. Clinical studies have explored LC’s impact on energy balance and weight loss. However, safety concerns have received limited attention [114]. A meta-analysis of 21 randomized, double-blind, and placebo-controlled studies lasting from 3 months to 1 year demonstrated that ALC improved cognitive dysfunction or slowed cognitive decline. The majority of trials used daily doses of 1.5–2.0 g of LC, which were well-tolerated [115]. In vitro and in vivo studies found no evidence of LC toxicity [4]. The observed safe level (OSL) of LC intake for chronic supplementation, according to the OSL risk assessment approach, is up to 2000 mg/day. Despite this, extensive research has been conducted on the potential benefits of using prophylactic doses of carnitine for various medical conditions. There is some debate and confusion regarding its use in normal nutrition. LC is non-toxic at oral doses up to several grams; therefore, supplements are often recommended for primary and secondary deficiencies. Supplemental intake of carnitine is generally well tolerated, as it is easily excreted. Both animal and human research data suggest potential health benefits when used as a medication. Patients with severe injuries, especially those with traumatic brain injury (TBI) and those with an estimated glomerular filtration rate (eGFR) above 94 mL/min/1.73 m^2^ have been found to have reduced plasma-free carnitine concentration [78]. Limited evidence exists for consuming LC exceeding 2000 mg/day [116]. However, no reports of LC intoxication exist, and healthy individuals have taken LC supplementation safely at doses ranging from 250 mg to 2.0 g per day [117].

## 6. Limitations, Conclusion, and Future Directions

The review thoroughly examined the various clinical interventions associated with LC in the management of TBI, AKI, and HA. The main goal was to highlight these interventions’ importance and identify all relevant preclinical and clinical studies with the utmost sensitivity. The use of LC in TBI, AKI, and HA has been extensively investigated, and its application in some conditions is progressing well, indicating its effectiveness in treating critical illnesses. However, challenges remain, such as the diverse presentations of TBI, AKI, and HA. The use of LC as an adjuvant therapy can also sometimes complicate clinical management. Furthermore, logistical barriers, including access to specialized care and healthcare disparities, must be addressed. In the studies reviewed, results for these critical illnesses were limited in both quantity and quality. The number of studies was insufficient, and the results were inconsistent. Overall, the trends in the effects were generally positive for many conditions. In some cases, LC has shown positive therapeutic effects, whereas in other scenarios, it has shown none. For instance, despite improvements in neurobehavioral function and the reduction of cerebral edema, a 7-day treatment with LC failed to reduce serum NSE levels or improve the mortality rate at 90 days in patients with TBI. There is low-quality evidence that the use of LC has no significant effect on reducing the 28-day mortality of septic shock patients. Although LC supplementation is an interesting approach that targets the metabolic imbalances of sepsis, its non-significant effect as a final result does not radically change the current standard of care for septic shock patients (AKI). LC administration has been found to improve impaired brain function in patients with liver cirrhosis. Muscle weakness and reduced exercise capacity can lead to a reduced quality of life, and LC is thought to have neuroprotective properties, as evidenced in animal models of cerebral ischemia and spinal cord injury [74,75]. However, current evidence does not fully support the use of carnitine supplementation in treating severe brain injuries and dialysis-related carnitine deficiency. Though carnitine supplementation may have a slightly positive impact on anemia-related markers, it appears to have little or no effect on adverse events. It is important to note that these conclusions are based on limited data and should be interpreted with caution. To reliably define the efficacy and safety of LC supplementation in patients requiring maintenance hemodialysis, high-quality randomized controlled trials with adequate sample sizes and hard endpoints are needed.

Preclinical and clinical studies have certain limitations. Small animal models offer advantages in studying TBI by allowing the screening of pathologies that are difficult to determine otherwise. The knowledge gained from findings in mice serves as a stimulus to explore concepts in higher mammals and humans. However, mouse models have limitations, including their small size and the lack of complexity of the mouse brain. Some conclusions drawn from animal studies may not directly translate to human patients. There is growing literature on the use of nutritionally based therapies for experimental brain injury treatment. However, these therapies face challenges similar to other treatment areas, such as the need for replication and verification of effects. This highlights the need for additional research to verify the effects in other brain injury models and under different laboratory conditions to assess how truly translational these therapies are. In a clinical setting, bias may arise due to study designs such as non-randomized controlled, randomized control, and retrospective analysis studies. Yahyapoor et al. (2023) are the first to discuss the simultaneous effects of LC on monitoring variables in TBI patients. The study reported some limitations such as low sample size, budget constraints, and difficulty accessing micronutrients in the blood. Additionally, precise weight and height measurements were infeasible due to ICU conditions [71]. Similarly, Mohammadi et al. (2017) reported limitations in their study involving AKI patients, including insufficient sample volume, unequal patient numbers in the two groups, lack of long-term patient follow-up, and difficulty in determining suitable doses of LC and its administration schedule. Future studies should consider other biomarkers and diagnostic factors of kidney damage and function to better understand the clinical benefits of LC’s efficacy against contrast-induced nephropathy (CIN) [97]. There is currently not enough evidence to support the use of LC supplementation for the treatment of dialysis-related carnitine deficiency. Though LC supplementation may have a slight impact on anemia-related markers, it does not seem to significantly affect adverse events. However, it is important to note that these conclusions are based on limited data and should be interpreted with caution [118].

There are some potential drawbacks and controversies surrounding LC supplementation. For example, LC is essential for lipolysis, the energy source during a hypercatabolic phase. However, LC is not present in nutritional replacement solutions. Additionally, free carnitine depletion, defined as a carnitine plasma level under 36 μmol/L, is not adequately reported in adult patients with severe trauma. Despite the important role of LC in the level regulation of acetyl-CoA, fatty acids, and nitrogen compounds, it should be noted that administration of LC alone cannot be applied in severe illnesses. L-carnitine should be considered as an adjuvant or a food supplement, but not as a medication. High dietary LC supplementation in humans or mice has a close association with cardiovascular disease risk. Short-term supplementation of LC is dose-dependently detrimental to isolated rat hearts before ischemia, as it is related to incomplete fatty acid β-oxidation. Carnitine interacts with pivalate-conjugated antibiotics, such as pivampicillin, which are used to prevent urinary tract infections. Chronic administration of these antibiotics can lead to carnitine depletion. However, although tissue carnitine levels in people who take these antibiotics may become low enough to limit fatty acid oxidation, no cases of illness due to LC deficiency in this population have been described. Treatment with the anticonvulsants valproic acid, phenobarbital, phenytoin, and carbamazepine reduces blood levels of carnitine [119]. The use of valproic acid with or without other anticonvulsants may cause hepatotoxicity and increase plasma ammonia concentrations, leading to encephalopathy. In most of our discussed studies focused on TBI, AKI, and HA, treatment with LC was found to be safe, well tolerated, and cost-effective. This makes it a drug worthy of further exploration for development and promotion. However, more randomized, controlled trials with different psychiatric and neurological disease designs are needed to fully assess its potential. Moreover, in the field of molecular biology, we still require a large number of studies to elucidate the specific mechanism of action of LC and explain its therapeutic effects on critical illnesses. This review emphasizes the necessity for ongoing research, innovation, and the establishment of standardized protocols to enhance the implementation of these interventions.

## Figures and Tables

**Table 1:** L-carnitine in health conditions other than TBI, AKI, and HA.

Medical Conditions	Effect	Produced Result
CVD/Peripheral Artery Disease	Transporting long-chain fatty acids in the myocardial mitochondria	Significantly reduces rates of all-cause mortality, ventricular arrhythmias, and new-onset angina but does not affect the risk of heart failure or myocardial reinfarction [64]
Insulin-Resistant Diabetes	Increases the removal of glucose from the bloodstream	Reduces fasting plasma glucose levels, increases high-density lipoproteins (HDL)-cholesterol, decreases triglycerides and insulin resistance, promotes whole-body glucose utilization, improves several lipid parameters, oxidative stress markers, and glycosylated hemoglobin (HbA1c) [65]
Infertility	Promotes sperm maturation, motility, and spermatogenesis. Promotes oocyte growth and maturation	Improves sperm motility and morphology [66] Greater ovulation rates and thicker endometrial tissue [67]
Osteoarthritis	Reduces levels of C-reactive protein	Decreases inflammation biomarkers and pain scores [68]
Athletic Performance	Preserves muscle glycogen and promotes fat oxidation	Increases aerobic fitness and endurance capacity [65]
Weight Loss	Cofactor for fatty acid oxidation	Increases weight loss [69]
Alzheimer’s Disease/Dementia	Acetylcholine synthesis and removal of toxic compounds to alleviate mitochondrial dysfunction	Improves cognitive impairment [70]

**Table 2:** Current clinical trials of LC for AKI treatment published by ClinicalTrials.gov [96] as of January 2024.

Title	Study ID	Phase	Other Conditions/Treatment
Acute Kidney Injury			
Preventing Contrast-induced Nephropathy: Evaluating Hydration Strategies and LC Administration	NCT01786824	Terminated	Renal insufficiency/hydration
Carnitine, Acylcarnitine, cell “Myocardial Function”, and CRRT	NCT01941823	Completed	Myocardial function
LC as an Adjunct Treatment for Septic Shock Patients With Acute Kidney Injury	NCT02664753	Recruiting	Septic shock

**Table 3:** Case studies on LC as a treatment for HA.

Study	Initial Ammonia (μmol/L)	Final Ammonia (μmol/L)	L-Carnitine
[101]	232	57	50 mg/kg/day QD by NG tube
[102]	348.8	58.13	4.5 g/day by IV
[103]	>700	50	2000 mg followed by 1625 mg q4h IV
[105]	Case 1: 101.5 Case 2: 101	Case 1: 28 Case 2: 22	1 g BID
[106]	377	47	100 mg/kg over 1 h IV
[107]	215	25	330 mg QID
[108]	Case 1: 110 Case 2: 65	Values not given, patients returned to baseline function	Case 1: 3 g IV Case 2: 3 g q8h IV
[109]	101	41	Not given
[110]	156	Normal, exact value not given	1 g q8h by NG tube

## Data Availability

Information for this review was collected from various sources including PubMed, Google Scholar, Scopus, Web of Science, and ClinicalTrials.gov.
